# 

**Published:** 1883-09

**Authors:** 


					﻿Whole Numbek,
CCCCLXIV.
September, 1883.
Number 3,
Vol. XLVII.

CHICAGO
MEDICAL
ournal & Examiner.
CHICAGO:
PUBLISHED BY THE CHICAGO MEDI-CAL PRESS ASSOCIATION
188 Clark Street.
®"Read the Notice of this Journal among Advertisements.
				

